# Implementing a pilot study of COVID-19 self-testing in high-risk populations and remote locations: results and lessons learnt

**DOI:** 10.1186/s12889-024-17930-2

**Published:** 2024-02-17

**Authors:** Elena Marbán-Castro, Vladimer Getia, Maia Alkhazashvili, Maia Japaridze, Ia Jikia, Berra Erkosar, Paula Del Rey-Puech, Guillermo Z. Martínez-Pérez, Paata Imnadze, Amiran Gamkrelidze, Olga Denisiuk, Elena Ivanova Reipold, Sonjelle Shilton

**Affiliations:** 1 FIND, Geneva, Switzerland; 2 National Center for Disease Control and Public Health (NCDC), Tbilisi, Georgia

**Keywords:** COVID-19, SARS-CoV-2, Self-testing, Diagnostic, Healthcare, School, Screening, Testing, Implementation

## Abstract

**Background:**

Rapid antigen-detection tests for SARS-CoV-2 self-testing represent a useful tool for pandemic control and expanding access to community-level case screening. COVID-19 self-tests have been extensively used in high-income countries since 2021; however, their introduction and programmatic implementation in low- and middle-income countries was delayed. We aimed to identify and continuously improve a weekly COVID-19 self-testing model among staff at healthcare facilities and schools.

**Methods:**

This mixed-methods, observational prospective study was conducted in 5 healthcare centres and 24 schools in Georgia, between June and December 2022. The study comprised the integration of COVID-19 self-testing into the national mandatory testing programme for high-risk groups, with primary distribution of self-tests among staff performed weekly, plus secondary distribution to their household members. These use cases were selected because NCDC was seeking to strengthen their already strong weekly testing programme, by investigating self-testing to ease the burden of testing in the healthcare system. Online surveys and semi-structured interviews were used for data collection.

**Results:**

In total, 2156 participants were enrolled (1963 female, 72%). At baseline and mid- and end-points, 88%, 97% and 99%, respectively, of participants agreed/strongly agreed they would self-test. Similarly, the majority were willing to report their self-testing results (88%, 98% and 96% at baseline and mid- and end-points, respectively). Weekly reporting of test results to the national COVID-19 database was high during all the implementation. There were 622 COVID-19 positive results reported, and linked to care, from 601 individuals (282 participants and 319 household members). Findings from qualitative interviews showed great satisfaction with self-testing for its convenience, ease of use, trust in the results, no need to travel for diagnostics, and increased perception of safety.

**Conclusions:**

Our findings contribute to the evidence-base regarding self-testing strategies conducted via workplaces and secondary distribution to households. Willingness to perform a COVID-19 self-test increased after implementation. This pilot enhanced pandemic preparedness through expansion of the national self-testing reporting system, development of communications materials, changes in the national legal framework and coordination mechanisms, and improved perceptions around self-care in the community. The lessons learnt can inform operational aspects of the introduction and scale-up of self-care strategies.

**Supplementary Information:**

The online version contains supplementary material available at 10.1186/s12889-024-17930-2.

## Background

The novel coronavirus disease 2019 (COVID-19) pandemic was one of the greatest challenges to public health in recent history. COVID-19 is caused by severe acute respiratory syndrome coronavirus 2 (SARS-CoV-2). Prevention of COVID-19 is a key control strategy that requires early and accurate diagnosis and prompt isolation of cases [1]. Screening and testing are cost-effective measures for COVID-19 control, as they enable contact-tracing that helps to ensure individuals isolate during their infectious period and accelerate these individuals’ access to psychosocial and clinical care [2–4]. In high-transmission scenarios, weekly testing for COVID-19 is a cost-effective strategy [5]. The World Health Organization (WHO) Strategic Preparedness and Response Plan for COVID-19 emphasised the need to accelerate equitable access to new tools to tackle COVID-19, including diagnostics, to reduce exposure, empower communities and protect the vulnerable [6].

In March 2022, WHO released interim guidance with a strong recommendation to use SARS-CoV-2 rapid antigen diagnostic tests (RADTs) as self-tests either as a diagnostic or a screening tool, depending on the epidemiological situation, appearance of symptoms, or recent exposure, and to facilitate linkage-to-care [7]. RADTs for self-testing allow individuals to fully perform tests themselves, from self-sampling to the interpretation of results; this may take place in an unsupervised or a supervised environment [8, 9]. Self-testing has particular benefits in limited-resource settings, as it does not require laboratory capacities, and it reduces the burden on the healthcare system as individuals can check their infection status without the need to attend a healthcare facility [10]. The WHO guidance highlights that in certain settings, such as schools and workplaces, serial COVID-19 self-testing may be recommended for the early detection of outbreaks [7]. Self-testing has been used worldwide to expand access to human immunodeficiency virus (HIV) diagnosis, especially in the most vulnerable populations [11–13]. In 2016, WHO recommended HIV self-testing as a safe, accurate and effective way to reach those in need of diagnosis [14]; in 2021, similar recommendations were made for hepatitis C virus (HCV) diagnosis [14, 15].

Since early 2021, COVID-19 self-tests have been extensively used in high-income countries, with many such countries deploying a wide range of self-testing strategies to complement testing efforts across multiple user-segments, including the workplace, schools, mass gatherings and the wider community [16, 17]. However, the introduction and programmatic implementation of COVID-19 self-tests in low- and middle-income countries (LMICs) was delayed by several months. This lag further contributed to the gap in access to COVID-19 testing between high-income countries and LMICs, exemplified by the inequities in testing rates across income groups. During the first quarter of 2021, daily testing rates per 1000 individuals in high-income countries were approximately 90 times higher than in low-income countries and approximately 11 times higher than in lower middle-income countries [18].

These inequities in access to testing during the COVID-19 pandemic highlight the importance of developing tools and models for the timely adoption and roll-out of self-testing that can be tailored to specific user-segments in LMICs. WHO stressed the importance of identifying optimal approaches to deliver SARS-CoV-2 self-tests, based on epidemiology, identified gaps in testing, and the broader response resources needed for prioritised population groups [7]. Any service delivery approach must be sufficiently agile to reflect the evolving epidemiology and be adaptable to suit the needs of the local public health system and community preferences [7]. Creating these strategies now can contribute towards pandemic preparedness efforts for other respiratory viruses, particularly in LMICs, and play a critical role in reducing the response time in the future.

This study forms part of a portfolio of projects led by FIND and multiple partners, which has included the implementation of COVID-19 screening models assisted by the distribution of SARS-CoV-2 self-test devices, in Brazil, Georgia, India, Malaysia and Viet Nam. This implementation research was designed to optimise and tailor SARS-CoV-2 self-testing approaches to specific contexts and defined user-segments, to improve service delivery models. Furthermore, this study was designed to be performed in Georgia, based on (i) geographical representation from different regions with different degrees of SARS-Cov-2 self-testing maturity; (ii) the importance of obtaining data from a country in the WHO European region, where there is a dearth of information relating to the feasibility of SARS-CoV-2 self-testing to increase case-detection rates; (iii) the availability of local partners with an interest in integrating SARS-CoV-2 self-testing as part of a national testing strategy; and (iv) previous experience of research into self-testing for other infectious diseases by the organisations implementing the study (FIND and the Georgia National Center for Disease Control and Public Health (NCDC)).

Georgia is an upper middle-income country, located at the intersection of Europe and Asia, and has a population of 3.7 million people [19]. Between the beginning of the pandemic in January 2020, to October 2022, the country reported more than 1.8 million COVID-19 cases [20, 21]. Since the start of the pandemic, the Georgian government has developed specific public health measures that were focused on identifying cases and preventing the spread of the virus [22]. Several interventions were implemented to reduce viral transmission, including a pass for vaccinated people or those who had proof of a negative COVID-19 test, and there was regular and frequent testing for high-risk groups, initially by reverse transcription polymerase chain reaction (RT-PCR) (since November 2020) and subsequently using RADTs, since February 2022 [23]. In Georgia, the first self-test for the detection of SARS-CoV-2 was authorised for use in April 2022; however, self-tests were still not being delivered to the public by the Georgian health system [24]. Regular, mandatory testing was provided for high-risk groups until May 2023; since then, mandatory testing has been replaced by a “recommendation” to test.

Populations at high risk of exposure to SARS-CoV-2 and/or who have difficulty accessing COVID-19 testing facilities, such as those living in remote, mountainous areas, would benefit most from self-testing strategies, as they do not require the travelling time and financial effort needed to access conventional, professionally delivered COVID-19 testing.

To introduce and scale-up self-testing in a particular setting or target population, it is essential to tailor and optimise the distribution model used. With this study, we aimed to identify and continuously improve SARS-CoV-2 self-testing strategies and supportive packages of a weekly self-testing model among staff at healthcare facilities and schools. The results would then be used to inform the potential integration of self-testing as part of the Georgian national SARS-CoV-2 screening programme for high-risk groups. Our study assessed a screening model among workplace staff and their household members, considering two important use cases: staff at high risk of exposure (hospital, clinic, nursing home and school staff) and staff in remote areas with limited access to healthcare (e.g. school staff in the Svaneti region). These use cases were selected because NCDC was seeking to strengthen their already strong weekly testing programme, by investigating self-testing to ease burden of testing in the healthcare system. The specific objectives were to: (1) assess the feasibility of a weekly SARS-CoV-2 self-testing model by examining the process, logistics, and site capacity to report self-testing results; (2) assess self-testing uptake and the reporting of results among staff and household members; (3) assess linkage-to-care following the use of a self-test; (4) assess knowledge acquisition with regards to self-testing; and to (5) explore participants’ satisfaction with the model. The primary outcomes of the analysis were perceptions around individuals’ willingness to self-test and report their COVID-19 self-test results to the national database.

## Methods

### Study design

This was a mixed-methods, prospective study conducted in a diverse range of working environments in Georgia between June and December 2022. These working environments comprised two hospitals, one clinic and one nursing home in Tbilisi; one nursing home in Kutaisi; and 24 public schools in the Svaneti region. In each location, a novel COVID-19 screening model was implemented, supported by the distribution of SARS-CoV-2 self-tests. The model included primary distribution of self-tests among staff at participating sites; these self-tests were to be performed weekly, if a staff member had symptoms, or if they were a contact of a person diagnosed with COVID-19. The model also included secondary distribution of self-tests, via staff members, to their household members and the wider community, to be used for individuals who were symptomatic or were a contact of a case. Self-tests were distributed free of charge to staff and their household members. This study was flexible in its design, to allow continuous improvements based on the results and feedback from staff members and stakeholders involved in the pilot study.

Key components of the self-testing package were communications materials and the system for reporting results. Communications materials were designed in collaboration with NCDC colleagues, to target knowledge gaps identified during the formative phase. These materials included a brochure and frequently asked questions (FAQs) for pilot participants, in addition to an existing video provided by the manufacturer of the self-tests.

### Formative phase and study periods

**Fig. 1:**
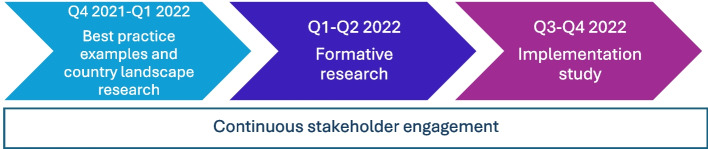
Timeline of the study’s design and implementation

Prior to launching the model in the targeted workplaces, a needs assessment and gap analysis phase was conducted, between October 2021 and January 2022. The purpose of this phase was to identify examples of best practice, conduct landscape research, perform stakeholder mapping and engagement, and define user segments. From January 2022 to May 2022, a formative research phase was conducted to inform the design of the screening model and the creation of the protocol and data collection tools. Implementation took place from June 2022 to December 2022, including trainings, screening, obtaining participants’ written informed consent, distribution of self-tests, and data collection. The overarching framework for this study (Fig. 1) was co-developed by FIND and NCDC.

### Study settings

NCDC selected all sites for participation during the needs assessment and gap analysis phase. The first criterion for selecting user segments and the sites was related to exposure, i.e. high-risk population groups who fell within the government’s mandatory COVID-19 screening programmes (weekly testing). The individuals who did not consent to enrol as participants in this study were advised to comply with government recommendations for healthcare provider-administered COVID-19 testing.

Tbilisi is the country’s capital and the most populous city in Georgia. Located in central-east Georgia, it has healthcare centres with some of the largest catchments in the country. The two largest government-run nursing homes in Georgia, located in Tbilisi and Kutaisi, were selected because of their high-risk populations (the elderly). Kutaisi, located in western Georgia, is one of the oldest cities in the country and the third-most populous.

The second criterion for selecting user segments and the sites was a lack of access to conventional SARS-CoV-2 testing due to geographical barriers. The Svaneti region, in Mestia district, is a mountainous area in the north-west of Georgia, where for most people there is no medical service within a radius of 30 km. Access to health resources in this area is very difficult, especially during the winter, due to heavy snow and transportation difficulties.

### Study participants and recruitment criteria

Study staff were recruited from personnel already working at the sites and who were then trained to help with the study procedures. Study staff were responsible for engaging potential participants in the study, training them in the use of self-tests, distributing self-tests, receiving results and reporting results to the Georgia NCDC national COVID-19 laboratory (LabCov) database. Participants were responsible for reporting their own results and those of any household members to study staff.

All staff from the selected sites were invited to participate in the study. After signing an informed consent form, they became participants. Participants were able to invite members of their household to participate in the study if they needed to self-test. The inclusion criteria included: willingness to provide informed consent, being aged more than 17 years, working or volunteering at the study sites, and willingness to self-test on a weekly basis. Age restrictions did not apply for household members. Minors were required to provide their assent in addition to the consent of their parents or legal guardians. No data were directly collected from household members (Table 1). The selection of participants for qualitative interviews was based on purposive convenience criteria to shortlist potential interviewees among the sample of participants. During the purposive sampling process, efforts were made to include participants from various genders, sites, job professions, and other relevant factors. Shortlisted participants were approached by staff at sites, in person or by phone to be invited to participate in a SSI.

**Table 1:**
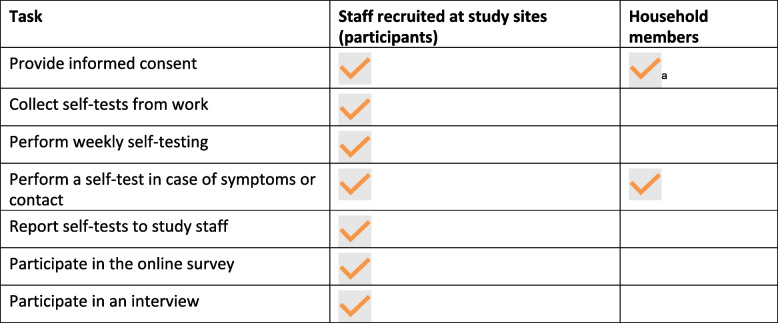
Overview of participants’ tasks

^a^When written informed consent could not be obtained, oral consent was obtained

### Implementation strategy

All recruited staff performed self-testing weekly, or more frequently in the case of symptoms or being a contact of a case, until 31 December 2022. Their household members used self-testing as needed if they were symptomatic or a contact of a case. As per the national guidelines, if a self-test result was negative, but the user had symptoms, they were advised to repeat a self-test after three days. If the result was positive, participants were asked to self-isolate. Participants knew their COVID-19 status within 15 min after self-testing and learnt to act accordingly. Counselling was provided, and all positive cases were linked to care according to the national guidelines. The internal channel used to communicate self-test results varied according to participants’ and study staff preferences, as well as the result (a positive result required instant communication). Reporting channels included phone calls, SMS, instant social messaging (such as WhatsApp®), a shared registry in Google Docs®, a paper-based sheet at work, and others. Participants could choose which channel to use to report their results.

The self-testing devices distributed were approved for use in Georgia and were considered to expose individuals to minimal risks. The COVID-19 self-test device used was the OnSite® COVID-19 Ag Self-Test (CTK Biotech, California, USA). The instructions for use were translated into Georgian by the manufacturer, reviewed by national stakeholders and optimised by potential participants based on cognitive interviews conducted during the formative phase.

### Data collection and processing

**Fig. 2:**
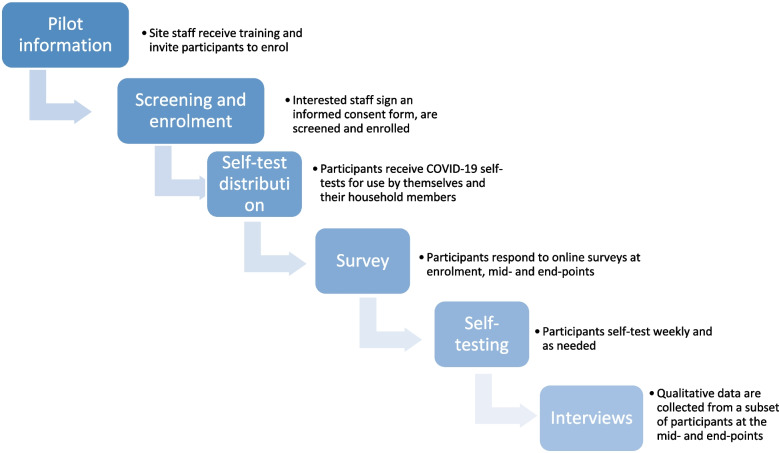
Flow diagram of study activities

The data collected included participants’ data regarding self-test use, participants’ responses to the online survey questionnaire, and qualitative data generated during the semi-structured interviews (SSIs) (Fig. 2).

#### Reporting data

COVID-19 self-test reporting data were collected in a study report log created in Microsoft Excel® (date, location, reason for performing the self-test, symptoms, and self-test result). Data from the reporting log were entered into the national LabCov database and validated according to NCDC database rules. National guidelines for linkage to care and surveillance procedures were followed for all results and all cases. Participants were responsible for reporting their household members’ self-test results to study staff. For the reporting of results, the NCDC and the Information Technology Agency (ITA) expanded the existing national LabCov database to incorporate COVID-19 self-testing results. In parallel, the national e-Health mobile application was expanded to incorporate reporting of self-testing results and was launched in the last weeks of the pilot study as an additional channel via which results could be reported. All results were linked to the existing COVID-19 national surveillance and care cascades. Cases that were positive by self-testing were tracked and managed according to the national protocol (reported to the Georgia Ministry of Health (MoH), to provide information about COVID-19 transmission trends and potentially lead to updates in national guidelines).

#### Surveys at enrolment, mid- and end-points

Alongside the implementation, an anonymous longitudinal online survey questionnaire was administered, to assess acceptability, knowledge and satisfaction. Participants were asked to complete the questionnaire at enrolment, mid-way through and at the end of the study, (Supporting information, Annex I; this version has been edited for consistency and to facilitate readers’ understanding). The questionnaire was written in English, based on insights gathered during the formative phase, and then translated into Georgian. The online surveys were continuously monitored for validity, including review by local stakeholders and responses from participants during the formative phase and during study implementation itself. The questionnaire was set up using the online data collection software, Alchemer®. All questions were mandatory, to minimise the risk of missing data. Data cleaning was performed using the Python programming language (version 3.9.7) integrated in Jupyter Notebooks and managed using the Visual Studio Code development environment (version 1.79.2). The database handling library used was “pandas” version 1.3.4. Data manipulation techniques were employed to enhance data quality and facilitate the integration of the three survey databases (at the baseline and mid- and end-points. Text substitution was performed using the “replace” function from “pandas”, to avoid misspelling or errors due to the manual input of data. Initial data cleaning involved rectifying incorrect identification numbers (“ID numbers”) to ensure consistency. Responses that lacked identifiable unique ID numbers were excluded from the analysis to maintain data integrity and reliability. Free-text responses collected in Georgian were translated into English. Subsequently, the three databases were merged using the unique ID numbers.

#### Qualitative data

Three female interviewers conducted the SSIs (EM-C, IA and NB). Two were medical doctors (MDs) and one held a PhD. All were part of the study team, two of them working exclusively for the study and one MD working at a public health hospital. One MD had no previous experience of interviewing but was trained to conduct interviews and had existing relationships with the interviewees. The other two interviewers had previous experience of conducting interviews, but no existing relationships with the interviewees. All interviewees knew about the study before the interviews took place. For the SSIs, study staff invited participants (staff at sites and also those who had a role as study staff) to be interviewed, until the saturation point was reached [25]. Interviewers’ were trained to minimized biasing participants responses while asking the questions, and to be aware of their internal biases.

The qualitative data collected were used to explore participants’ perceptions of and experiences with the self-testing model, their success stories, and any potential social harms. Interviews were performed online, with participants at their homes or at any place decided by them, thus, potentially other people were present beside participants and researchers. The interviews lasted approximately 30 min, were audio-recorded and notes were taken. The interviewers used a guide that was specifically developed for this study and can be found in the Supporting information (Supporting information, Annex II).

### Data analysis

The various datasets collected were analysed separately, as outlined below.

#### Reporting data

The self-test results collected in the reporting logs were monitored using an Excel tool. The data were cleaned and analysed using R 4.2.2 statistical software. Descriptive statistics were calculated using the “vtable” package, “sumtable” function in R. The uptake and reporting of self-tests to the national database were analysed by sex at birth, site, type of user and self-test result.

#### Surveys at enrolment and the mid- and end-points

The descriptive statistics were calculated as described above for the reporting data. Linear regression analysis was conducted to investigate relationships between willingness to self-test and to report results and perceptions around being worried about COVID-19 and understanding the benefits of self-testing. Responses were recorded using a five-point Likert scale and were coded from –2 (“strongly disagree”) to 2 (“strongly agree”), with 0 being “neutral”; time was coded as 1 (baseline), 2 (mid-point) and 3 (end-point). The regressions were built using general linear models using the “lm” function in the “stats” package. Contrasts were defined using the “emmeans” function within the “emmeans” package. Visualisations were created using “ggplot2”. *P*-values were adjusted for multiple comparisons using "p.adjust” function in R and using Benjamini-Hochberg (BH) method. Due to the large sample size, the significance threshold for the *p*-value was defined as 0.001.

#### Qualitative data

The SSIs included a set of questions that corresponded to (1) sociodemographic context and previous experiences with COVID-19, (2) perceptions and satisfaction with self-testing, (3) use of self-tests and value of the reporting mechanism, and 4) exploratory questions about willingness to pay for a COVID-19 self-test. The SSIs were audio-recorded, and notes were taken. Thematic analysis was conducted [26]. Meetings were held with the study team to identify and discuss common themes and codes. The coding process involved a combination of inductive and deductive approaches, with pre-existing concepts from the interview guide used to categorise the information (themes were deductive from the guide) and codes derived from the data (inductive codes). COREQ guidelines were followed (Supporting information, Annex III) [27].

### Ethics considerations and approval

The main risk that could derived from participation in the study would be social harm resulting from a breach in confidentiality. To minimise this risk and prevent its occurrence, study staff at all study sites were trained in ethics and confidentiality issues. All participants provided written informed consent. Household members provided oral consent when written informed consent could not be obtained. This study protocol was approved by the NCDC Institutional Review Board (Ref.: # 2022-049, May 24, 2022). The study was conducted in accordance with the Belmont Report [28], the Declaration of Helsinki [29] and applicable ICH Good Clinical Practice E6 (R2) standards [30].

## Results

### Screening, enrolment, refusal to participate and withdrawal

**Fig. 3:**
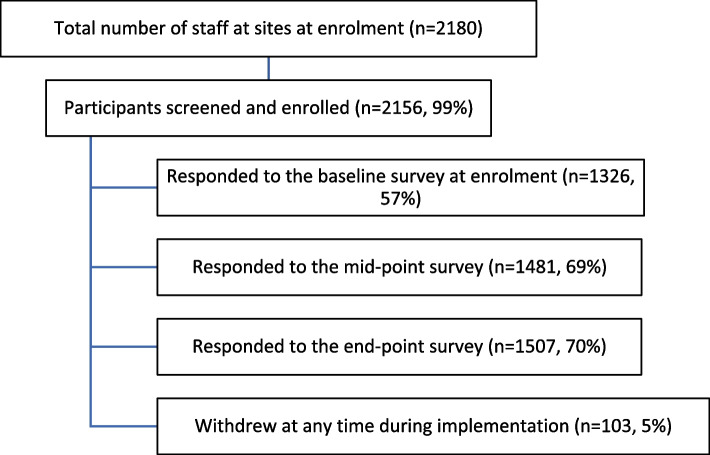
Participant numbers and survey response proportions

A total of 2156 staff from the various sites were enrolled in the pilot study, which corresponded to 99% of the total number of staff (Fig. 3 and Table 2). Smaller sites (< 100 employees) enrolled 100% of their staff. Overall, just 19 participants refused to enrol, and 103 withdrew during the implementation. In addition, 582 household members were enrolled during the implementation of the pilot study, yielding a total of 2738 self-test users.

**Table 2:** Enrolment, refusal and withdrawal rates, by site and overall

	**Site**						
	**1**	**2**	**3**	**4**	**5**	**6**	**Total**
**Total staff**	937	340	285	48	70	500	2180
**Enrolment, n (% among total staff)**	928 (99%)	334 (98%)	280 (98%)	48 (100%)	70 (100%)	496 (99%)	2156
**Refusal, n (% among total staff)**	6 (0,6%)	4 (1,2%)	1 (0,4%)	0	0	8 (1,6%)	19
**Withdrawal, n (% among enrolled participants)**	44 (5%)	4 (1%)	33 (12%)	2 (4%)	6 (9%)	14 (3%)	103

Sites 1, 2 and 3 were healthcare centres, sites 4 and 5 were nursing homes, and site 6 corresponded to 24 schools

Table 3 shows the participants’ basic sociodemographic data and household members who were screened and enrolled. Most participants (71.7%) were female, with the majority aged less than 31 years (25.5%) or aged 51 to 60 years (21.4%). Of all self-testing users, 20% were household members (Table 3).

**Table 3:** Baseline demographic characteristics of participants and household members screened and enrolled, by site and overall

**Type of site**	**Characteristic**	**n**	**Percentage**
**Healthcare centre**	**Type of user**	*N* = 2075	
	Participant (staff enrolled)	1542	74.3%
	Household member	533	25.7%
	**Gender**		
	Female	1454	70.1%
	Male	621	29.9%
	**Age**		
	< 31	631	30.4%
	31-40	321	15.5%
	41-50	317	15.3%
	51-60	390	18.8%
	≥ 61	416	20%
**Nursing home**	**Type of user**	N = 132	
	Participant (staff enrolled)	118	89.4%
	Household member	14	10.6%
	**Gender**		
	Female	104	78.8%
	Male	28	21.2%
	**Age**		
	< 31	15	11.4%
	31-40	20	15.2%
	41-50	30	22.7%
	5160	40	30.3%
	≥ 61	27	20.5%
**School**	**Type of user**	N = 531	
	Participant (staff enrolled)	496	93.4%
	Household member	35	6.6%
	**Gender**		
	Female	405	76.3%
	Male	126	23.7%
	**Age**		
	< 31	52	9.8%
	31-40	103	19.4%
	41-50	124	23.4%
	51-60	155	29.2%
	≥ 61	97	18.3%
**Overall**	**Type of user**	*N* = 2738	
	Participant (staff enrolled)	2156	78.7%
	Household member	582	21.3%
	**Gender**		
	Female	1963	71.7%
	Male	775	28.3%
	**Age**		
	< 31^a^	698^a^	25.5%
	31-40	444	16.2%
	41-50	471	17.2%
	51-60	585	21.4%
	≥ 61	540	19.7%

^a^This number of household members includes 116 children and adolescents (< 18 years old)

### Sociodemographic characteristics of participants

More detailed sociodemographic data were collected from participants who completed the online enrolment survey (57% of those enrolled) (Table 4). Among the participants, 80% self-identified as female, and the mean age was 47.2 years. Regarding educational background, 65% of participants had completed university studies. Participants from healthcare centres and schools had a higher level of education compared with participants from nursing homes (67% and 69% university level vs. 39%, respectively). More than 20% of participants at all sites lived with four or more household members, but for the majority just one of them had been employed in the past three months. Smartphone ownership was lower among participants from schools in the Svaneti region (65%) compared with participants from healthcare centres in Tbilisi (86%) and nursing homes in Tbilisi and Kutaisi (87%). Most participants from the schools in Svaneti had not received any dose of COVID-19 vaccine (67%). More than half of participants at all sites had been previously diagnosed with COVID-19, especially those from nursing homes (84%). While most participants from healthcare centres (77.2%) and schools (83.3%) declared they had no medical condition/risk factors for COVID-19, just 2.6% of participants from nursing homes declared they had no medical condition/risk factors for COVID-19.

**Table 4:** Sociodemographic characteristics of participants

	**Type of site**							
**Variable**	Healthcare centre		Nursing home		School		Overall	
	*N* = 823		*N* = 116		*N* = 366		*N* = 1305	
Age (mean, standard deviation)	46.5 (15.4)		47.7 (13.0)		48.8 (12.7)		47.2 (14.5)	
	n	%	n	%	n	%	n	%
**Gender**								
Female	657	79.8%	98	84.5%	290	79.2%	1045	80.1%
Male	165	20.0%	18	15.5%	76	20.8%	259	19.8%
Prefer not to say	1	0.1%	0	0%	0	0%	1	0.1%
**Education**								
Elementary school	0	0%	1	0.9%	1	0.3%	2	0.2%
High school	58	7.0%	17	14.7%	48	13.1%	123	9.4%
Professional technical education	203	24.7%	52	44.8%	63	17.2%	318	24.4%
Secondary school	7	0.9%	1	0.9%	1	0.3%	9	0.7%
University (bachelor’s or master’s degree)	529	64.3%	44	37.9%	251	68.6%	824	63.1%
University (PhD)	26	3.2%	1	0.9%	2	0.5%	29	2.2%
**Occupation**								
Administration	27	3.3%	1	0.9%	4	1.1%	32	2.5%
Assistant	0	0%	0	0%	1	0.3%	1	0.1%
Cleaner	20	2.4%	6	5.2%	25	6.8%	51	3.9%
Cook	0	0%	3	2.6%	0	0%	3	0.2%
Director	7	0.9%	2	1.7%	20	5.5%	29	2.2%
Driver	7	0.9%	1	0.9%	7	1.9%	15	1.1%
Laboratory personnel	11	1.3%	0	0%	0	0%	11	0.8%
Librarian	1	0.1%	0	0%	8	2.2%	9	0.7%
Maintenance staff	77	9.4%	8	6.9%	26	7.1%	111	8.5%
Manager	0	0%	0	0%	1	0.3%	1	0.1%
Medical doctor	314	38.2%	13	11.2%	3	0.8%	330	25.3%
Nurse	228	27.7%	25	21.6%	6	1.6%	259	19.8%
Other	80	9.7%	50	43.1%	15	4.1%	145	11.1%
Sanitarian	51	6.2%	7	6.0%	0	0%	58	4.4%
Social worker	0	0%	0	0%	1	0.3%	1	0.1%
Teacher	0	0%	0	0%	249	68.0%	249	19.1%
**Household members**^a^								
0	95	11.5%	16	13.8%	25	6.8%	136	10.4%
1	175	21.3%	24	20.7%	56	15.3%	255	19.5%
2	205	24.9%	26	22.4%	60	16.4%	291	22.3%
3	180	21.9%	30	25.9%	87	23.8%	297	22.8%
≥ 4	168	20.4%	20	17.2%	138	37.7%	326	25.0%
**Household members employed in the past 3 months**								
0	201	24.4%	33	28.4%	132	36.1%	366	28.0%
1	352	42.8%	44	37.9%	173	47.3%	569	43.6%
2	180	21.9%	26	22.4%	42	11.5%	248	19.0%
3	66	8%	11	9,5%	13	3,6%	90	6,9%
≥ 4	24	2.9%	2	1.7%	6	1.6%	32	2.5%
**Children under 12 years in the household**								
0	490	68.1%	69	69.0%	211	61.9%	770	66.3%
1	133	18.5%	15	15.0%	60	17.6%	208	17.9%
2	79	11.0%	16	16.0%	45	13.2%	140	12.1%
3	13	1.8%	0	0%	20	5.9%	33	2.8%
≥ 4	5	0.7%	0	0%	5	1.5%	10	0.9%
**Smartphone ownership**								
No	113	13.7%	15	12.9%	127	34.7%	255	19.5%
Yes	710	86.3%	101	87.1%	239	65.3%	1050	80.5%
**COVID-19 vaccination doses received**								
0	108	13.1%	52	44.8%	244	66.7%	404	31.0%
1	26	3.2%	6	5.2%	6	1.6%	38	2.9%
2	404	49.1%	46	39.7%	109	29.8%	559	42.8%
≥ 3	285	34.6%	12	10.3%	7	1.9%	304	23.3%
**Previous diagnosis of COVID-19**								
Yes	638	77.5%	97	83.6%	188	51.4%	923	70.7%
No	166	20.2%	19	16.4%	176	48.1%	361	27.7%
Don’t know/remember	19	2.3%	0	0%	2	0.5%	21	1.6%
**Previous COVID-19 severity of symptoms**								
Asymptomatic	83	13.2%	13	13.5%	6	3.2%	102	11.1%
Flu-like symptoms	311	49.3%	22	22.9%	64	34.0%	397	43.4%
Mild to moderate symptoms	216	34.2%	52	54.2%	100	53.2%	368	40.2%
Severe symptoms	21	3.3%	9	9.4%	18	9.6%	48	5.2%
**Previous medical conditions/risk factors**^b^								
None	635	77.2%	3	2.6%	305	83.3%	1034	79.2%

^a^Excluding participants

^b^Chronic conditions (cancer; heart condition, stroke, or cerebrovascular disease; immunocompromised condition; cystic fibrosis; chronic lung, kidney, or liver disease; transplant etc.), Infections (HIV, tuberculosis, others), Diabetes, overweight, physical inactivity, Mental health/neurological conditions (schizophrenia spectrum disorder, depression, dementia, others), Disabilities, Pregnancy, Smoking (current or former), and Substance use disorder

### COVID-19 self-testing reporting

A total of 52,985 self-tests were reported to the national COVID-19 database (Table 5). Of these self-tests, 41,443 (78%) were performed by females. Just 3.3% of COVID-19 self-tests were performed in the presence of symptoms. There were 622 COVID-19 positive results reported from 601 individuals (282 participants and 319 household members). A total of 1080 self-tests were used and reported by household members, of which 31% were positive. The majority of COVID-19 self-tests (95%) were performed in households.

**Table 5:** Number of COVID-19 self-tests reported to the national database by type of user, gender, presence of symptoms, self-test result and location of use

**Variable**	**n**	**%**
**Type of user**	*N* = 52,985	
Participant	51,905	98%
Household member	1080	2.0%
**Gender**	*N* = 52,985	
Female	41,443	78.2%
Male	11,542	21.8%
**Presence of symptoms**	*N* = 52,555	
No	50,812	96.7%
Yes	1743	3.3%
**COVID-19 self-test result**	*N* = 52,985	
Invalid	60	0.1%
Negative	52,303	98.7%
Positive	622	1.2%
**Location of COVID-19 self-test performed**	*N* = 52,985	
Home	50,346	95.0%
Work	2504	4.7%
Other	135	0.3%

More than 78% of all self-tests reported by participants were performed by females, compared to 35% to 47% of female household members (Supporting information, Annex IV). A significant percentage of household members tested positive, 29.4% in healthcare centres compared with 64.3% in nursing homes, and 51.4% in schools (Supporting information, Annex V). While most COVID-19 self-tests performed as part of the participants’ weekly monitoring were negative (97%), among all positive cases, 81% of them self-tested because they had symptoms (Table 6). Asymptomatic infections were detected in 19% of positive self-tests performed by participants and in 6.9% of those performed by household members (Table 6).

**Table 6:** Number of COVID-19 self-test results reported to the national COVID-19 database, by reason for testing and by participants and household members

**Self-test result**	**Reason for testing**	**Participants**			**Household members**		
		n	% among self-test by type of result	% among total self-tests	n	% among self-test by type of result	% among total self-tests
**Positive**	**Symptoms**	234	81.0%	0.5%	308	92.5%	58.1%
	**Contact**	9	3.1%	0%	23	6.9%	4.3%
	**Monitoring**	46	15.9%	0.1%	NA		
	**Other**	0	0%	0%	2	0.6%	0.4%
**Invalid**	**Symptoms**	4	7.4%	0%	5	83.3%	0.9%
	**Contact**	5	9.3%	0.0%	1	16.7%	0.2%
	**Monitoring**	45	83.3%	0.1%	NA		
	**Other**	0	0%	0%	0	0%	0%
**Negative**	**Symptoms**	647	1.3%	1.2%	606	82.6%	11.4%
	**Contact**	421	0.8%	0.8%	128	17.4%	24.1%
	**Monitoring**	50,401	97.7%	97.1%	NA		
	**Other**	100	0.2%	0.2%	0	0%	0%

*NA* not applicable

Since all study sites started enrolment, self-testing reporting rates, among participants and their household members, remained high among the 26 weeks of the implementation (Supporting information, Annex VI).

### COVID-19 perceptions, willingness to self-test and to report results

**Fig. 4:**
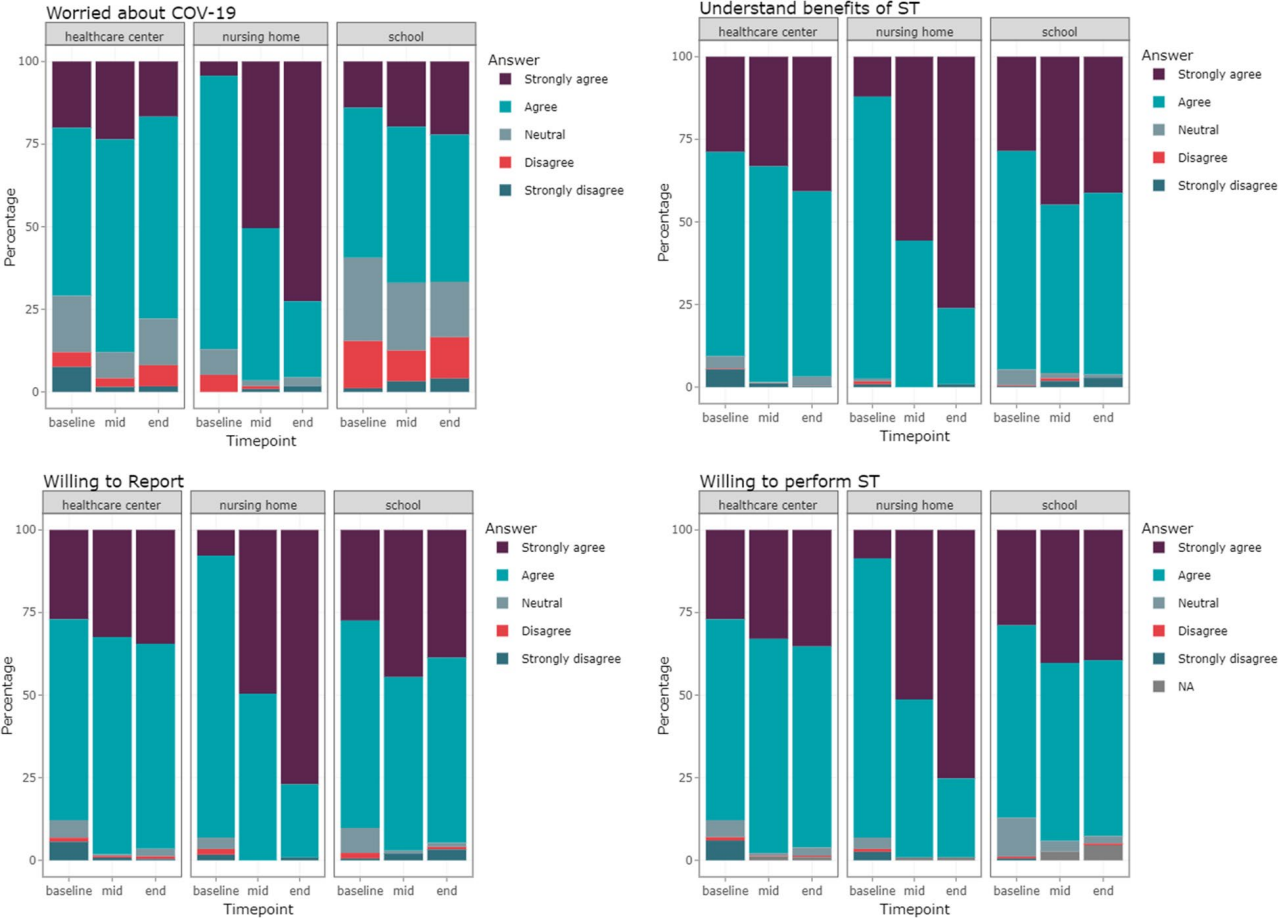
Reported perceptions about COVID-19 and willingness to self-test and report results, by site

Baseline and mid- and end-point online surveys were completed by 1326 (57%), 1481 (69%) and 1507 (70%) participants, respectively. Most participants at all sites and times agreed/strongly agreed with the idea of self-testing for SARS-CoV-2 (88% at baseline, 97% at mid- and 95% at end-point). Similarly, the majority of participants were willing to report their results after self-testing (88% at baseline, 98% at mid-point and 96% at end-point). Willingness to perform and report self-testing results increased during implementation, especially in nursing homes (Fig. 4).

Participants’ understanding of the benefits of COVID-19 self-testing increased during the implementation period. However, participants had differing levels of worry regarding COVID-19, at different times and across the various sites, with participants in nursing homes reporting being more worried about COVID-19 at the end-point (December 2022) (Supporting information, Annex VII). To have a deeper understanding of the trends in the perceptions over time in different type of sites, we have analysed the data using linear regressions (Supporting information, Annex VII). In all cases, main effects (Time and Site type) were significant overall (adjusted *p*-value < 001) highlighting a general increase over time, and site to site differences. However, the most obvious trend was the change of opinion in time that was different by site type: Worries about COVID-19, understanding the benefits of self-testing, willingness to report and willingness to perform a self-test highly increased over time in nursing homes but not necessarily in schools or health care centres. This was supported by a statistically significant *p*-value for the interaction term in all cases (adjusted *p*-value < 0.001 for all questions (Supporting information, Annex VII). Age and sex were not significant in any of the regressions.

### Knowledge about COVID-19 self-testing

Overall, there was an increase seen in the knowledge levels from the baseline data to the end-of-study data. At baseline, 80% of participants correctly answered where from their body they should take a sample for self-testing, compared with 91% at end of the intervention (Table 7). Regarding what a positive result from a self-test means, knowledge levels were very similar at the baseline (88%), mid- (92%) and end-points (89%) of the study. A correct understanding of what the faint line in a self-test cassette means was understood at baseline by 53% of participants, followed by 59% and 68% at the mid- and end-points, respectively. However, some knowledge gaps remained; for example, 20% of participants thought that after a positive result with a faint line, they would need to repeat a self-test.

**Table 7:** Knowledge about COVID-19 self-testing at baseline and mid- and end-points, among all participants

**Knowledge question**	**Answers**	**Baseline**		**Mid-point**		**End-point**	
		**n**	**%**	**n**	**%**	**n**	**%**
**Subtotals**		*N* = 1326		***N*** = 1481		*N* = 1507	
Where should you collect a sample from using a COVID-19 self-testing swab?	One nare, no more than 2-cm deep	29	2.19	21	1.42	11	0.73
	Mouth and nose	27	2.04	7	0.47	6	0.4
	Nose, does not matter how deep, but move the swab in circles	81	6.11	85	5.74	74	4.91
	Nose, more than 2-cm deep	125	9.43	85	5.74	45	2.99
	**Two nares, no more than 2-cm deep**	**1064**	**80.24**	**1283**	**86.63**	**1371**	**90.98**
What does a positive result mean?	**Likely to be infected with COVID-19**	**1168**	**88.08**	**1361**	**91.9**	**1346**	**89.32**
	Not infected with COVID-19	93	7.01	86	5.81	110	7.3
	Had COVID-19 in the past	42	3.17	24	1.62	27	1.79
	Have a high likelihood of developing severe symptoms	10	0.75	6	0.41	18	1.019
	Don’t know	13	0.98	4	0.27	6	0.4
What does the faint line in a COVID-19 self-test cassette mean?	Don’t have COVID-19	94	7.09	76	5.13	31	2.06
	Have COVID-19 but cannot infect others	61	4.60	127	8.58	120	7.96
	**Might have COVID-19 and can infect others**	**711**	**53.62**	**878**	**59.28**	**1021**	**67.75**
	Need to repeat the COVID-19 self-test	417	31.45	380	92.66	307	20.37
	False-positive result	43	3.24	20	1.35	28	1.86

Responses according to national guidelines are displayed in bold

### Key qualitative themes: participants’ perceptions and experiences

During the implementation pilot study, 54 SSIs were performed; most participants were female, aged more than 41 years and from healthcare centres. At the mid-point, 32 SSIs were performed, with most participants being female, aged 41 to 50 years and from healthcare centres (Supporting information, Annex VIII). At the end-point, 22 SSIs were performed, 16 with participants and 6 with participants who were also study staff. Most of the participants were female, aged more than 41 years and from healthcare centres. Themes identified in advance, following the interview guide included: previous experiences with COVID-19, COVID-19 self-testing experiences, advantages, disadvantages, feelings and willingness to pay for COVID-19 self-testing (Supporting information, Annex IX).

Among all participants interviewed, the most commonly reported advantages of COVID-19 self-testing were that it was comfortable/painless, time-saving, simple/easy and convenient. Only two people expressed concerns about self-testing, in interviews performed at the mid-point; these concerns were related to fears/doubts about other individuals not reporting their results. Most participants reported that it would be very valuable to have COVID-19 self-tests available for the general population, as exemplified by the following quote:

*“When people outside work heard about the project and self-test availability they were jealous, and wanted also to buy them”* (Female, 34-year-old, caregiver)

Participants shared how weekly self-testing made them feel. Most responses were related to feeling calm knowing that their work colleagues were being screened and were testing negative, and that they could easily access a simple self-test if they or their household members had symptoms or had been in contact someone who had COVID-19.

*“The project caused us peace”* (Female, 52-year-old, accountant)

Although it depends on the price, most participants reported they would be willing to buy COVID-19 self-tests if they were available in pharmacies or shops. In total, 45 participants responded to the question regarding the price they would pay for a self-test. Of them, 40% stated that the price should be approximately 5 lari (1.9 USD), 20% stated it should be from 5 to 10 lari (1.9 to 3.8 USD), with other participants stating prices that ranged from 0.5 to 30 lari. Participants from the Svaneti region and Kutaisi were less likely to identify a price compared with participants from Tbilisi city. The lowest price, 0.5 lari (0.2 USD), that would be paid for a self-test was stated by a participant from Svaneti, and the maximum price, 20 to 30 lari (7.7 to 11.6 USD), was stated by a participant from Tbilisi. Participants noted the need for the government to adapt the price of self-tests to ensure they were available to the most vulnerable populations, such as the elderly, those who cannot afford to pay for tests, school students etc.

*“Previously, school staff shared transportation to the testing location, and if one tested positive, all of us had to self-isolate”* (Female, 39-year-old, teacher)

By the end-point of the study, most participants’ perceptions about COVID-19 self-testing had changed in a positive way. Most of their concerns at the beginning were related to uncomfortable experiences during PCR or professional antigen testing. During the pilot study, the participants realised that the nasal swabs used for self-tests were very comfortable and painless. Furthermore, none of the participants reported any concerns regarding a lack of trust in their self-test results or any privacy issues regarding the handling of their data.

Questions about participants’ behaviour upon a receiving a positive COVID-19 result were only asked during the end-of-study interviews, to the final six participants. Three of them disclosed that they self-tested positive for COVID-19 during the pilot study, which enabled them to promptly self-isolate. Participants also related that on some occasion they had an invalid result, and they knew what to do, to call their assigned study staff and perform another self-test, as illustrated in the following quote.

“*One invalid result was yesterday, the test did not show any result, I was informed that this kind of case might happened, so I knew what to do, took another test and notified my facilitator accordingly*.” (Male, 19 years old, administrative)

Similarly, one participant shared that he had a faint line result in one self-test and knew what he needed to do, as he had no doubts about the result being positive:

“In 2022 July, I was positive, used ST, the line was very faint but for sure considered as a positive and self-isolated” (Male, 35 years old, medical doctor).

Six study staff were interviewed at the end of the study, not only to share their experiences as participants but also as study staff. No difficulties were reported in terms of building positive and trusting relationships with participants. Study staff recognised that their role in the pilot was essential for creating a welcoming environment, where participants felt comfortable sharing their self-testing results, concerns and experiences, and identifying any challenges so they could be appropriately addressed. Some study staff, especially those living in Svaneti, saw the value in self-testing and declared that they would continue helping staff at their site and other staff to enable self-testing to continue. This may have been due to their bad experiences with transportation for the previous testing method and their good experiences with self-testing. During implementation, self-testing increased participants’ perception of safety (at work and in their wider environment). At a mid-point workshop, preliminary results were shown to the study staff. As most positive cases were detected among those who self-tested when they had symptoms, discussions were held regarding whether self-testing should occur only in symptomatic individuals. However, due to the participants’ increased perception of safety, the study staff preferred to continue with weekly self-testing.

### Implementation results

For this study, several legal documents had to be signed by Georgia’s MoH to register, import and distribute COVID-19 self-tests in the country. The expansion of the existing reporting system for recording COVID-19 testing results (the LabCov database), to incorporate self-testing results, enabled the pilot study data to be integrated into the existing national data management and linkage-to-care systems. The platform was ready for use when the pilot study started in June 2022. An expansion of the national e-Health mobile application was carried out, with the engagement of the ITA, in parallel with the pilot study implementation and was launched in December 2022. Participants were able to directly upload their self-testing results with minimal data entry, and their results were reflected in LabCov in real-time. The e-Health application was initially rolled out among study staff and then expanded to the other participants in January 2023. Additionally, various communications materials were co-designed by FIND and NCDC to address specific knowledge gaps identified during the formative research phase. Communications materials that were provided to study staff during the train the trainer sessions included FAQs and training materials about the study’s procedures. Communications materials that were given to study participants by the study staff during the distribution of self-tests included FAQs, a brochure and a link to the manufacturer’s video. Additional gaps in knowledge identified during the pilot implementation and preliminary analysis of results, were addressed with workshops and trainings targeted for the specific sites. Additionally, four videos with information and case studies about self-testing were created, in the Georgian language, for use by the general population.

## Discussion

The present study aimed to assess and improve the distribution models used for self-testing for SARS-CoV-2 in healthcare centres and schools in Georgia and to generate data to inform the potential inclusion of self-testing as part of the national testing programme. We employed a mixed-methods, observational, prospective approach. The findings of this study have provided valuable insights into the feasibility and acceptability of self-testing as a strategy for COVID-19 control, particularly in workplace settings and in households of individuals at high risk of exposure and who are in remote areas. Additionally, the study has provided information about the operational aspects of implementing and scaling up self-testing in resource-limited settings.

Georgia has extensive experience in designing and implementing self-testing strategies for HIV and HCV. However, at the time this pilot study was initiated, COVID-19 self-tests were not widely available in the country and were not part of national policies. Despite the multiple differences in disease epidemiology and risk factors between these infections, Georgia’s previous experience in self-testing played a key role in the success of the implementation of this study.

Our results suggested that routine monitoring for COVID-19 using self-tests was feasible and acceptable among staff at healthcare and education centres. Depending on the epidemiological situation, and following updated guidelines, regular self-testing can be well-received and integrated into routine testing practices, enhancing case screening at the community level. Other studies have found similarly high acceptability of regular COVID-19 self-testing in various populations, including students [31], children at day care centres [32] and primary school children [33]. Our pilot study highlights the feasibility of using existing human resources and systems in place at both the site and country level to operationalise self-testing strategies.

The implementation of the self-testing pilot study was successful, as evidenced by the high enrolment rates and the substantial number of self-tests reported to the national COVID-19 database and thus linked to care. A high proportion of participants actively participated in the self-testing programme, with high weekly reporting rates during the 26 weeks of implementation. This indicates a high level of engagement with and adherence to the self-testing protocol, further supporting the feasibility of self-testing as a widespread screening strategy and the importance of providing flexibility in the reporting channels. While most positive cases were detected among symptomatic individuals (81%), our engagement with pilot study staff and other national stakeholders during the implementation revealed a preference for weekly testing, based on a corresponding increased perception of safety.

Notably, through the self-testing pilot study, a considerable number of COVID-19 positive cases were identified, both among participants and their household members. The detection of positive cases among participants and their household members highlights the potential of self-testing to identify infected individuals and facilitate timely linkage-to-care. This finding underscores the importance of self-testing for detecting and containing viral spread, especially in remote settings, among individuals who are far from a healthcare centre and employees at high risk of exposure. The low rate of detection of asymptomatic infections in this pilot (18% in participants and 7% in household members) suggests that a scaled-up program may benefit from targeted testing based on factors such as symptoms, exposure risk, and community case pressure, while there is not an epidemic peak. Further studies are needed to establish the cost-effectiveness of different testing strategies depending on the epidemic stage to optimize the best use of resources. The high proportion (31%) of cases detected among household members provides evidence of the importance of the secondary distribution of tests to further increase detection.

This study provides evidence for the importance of developing and tailoring self-testing support packages, in particular to increase knowledge and awareness about both testing and self-testing. The knowledge surveys we conducted indicated an increase in participants’ knowledge from the baseline to the end-point. This was likely due to the continuous provision of communications materials, the role of the study staff (who were always available to answer questions and address any doubts), and targeted discussions and trainings held to address specific knowledge gaps identified during implementation. The role of the study staff to be the contact point during the implementation for their assigned participants was key for participants to build confidence, to empower them to self-test, to solve concerns, and to explain when and who to self-test (for example, in case of household members with symptoms), and to understand their self-test results.

The success of the pilot study contributed to the expansion of self-testing in other healthcare centres and among the general population in different areas of the country. When the pilot started, self-testing was offered by NCDC as an additional testing requirement to professional testing. However, as evidence from the pilot study emerged and the severe phase of the COVID-19 pandemic was declared to be over, NCDC and the MoH decided to continue with the self-testing approach among those sites participating in the pilot study. Self-testing was also expanded to patients on dialysis, medical staff at emergency centres, etc. The pilot study provided valuable insights into the operational aspects of self-testing, informing the scale-up process and enabling NCDC to expand their national database for reporting self-testing results. The integration of self-testing into the national e-Health mobile application demonstrates the potential for technology-based solutions to enhance self-testing implementation and data management. Community-based asymptomatic testing has been associated with substantial reductions in COVID-19-related hospital admissions [34].

This study contributes to the evidence base on the use of self-testing in workplaces, particularly for staff at high risk of exposure, and the secondary distribution of self-tests to household members and the community. The findings highlight the acceptance by individuals of incorporating self-testing into comprehensive testing strategies during health emergencies, especially in resource-limited settings, and how self-testing can play a role in changing people’s mindset and culture around self-care, in a context of routine care.

The information resulting from the assessment of this enhanced screening model enabled the creation of self-testing implementation resources for Georgia and other public health resource-constrained countries. These resources can subsequently be used to rapidly deploy and scale-up self-testing strategies as part of pandemic preparedness. As there is a dearth of evidence on the costs of self-testing, as well as how to deploy and scale-up self-testing for outbreaks and health emergencies, the lessons learnt from this study can also inform self-testing modalities for future pandemics or for health emergencies that are endemic in many LMICs.

As suggested by qualitative studies conducted in Indonesia and Brazil [10, 35], the use of self-testing could reduce the demand on health facilities while addressing many of the usual barriers to the uptake of services, leading to more timely testing of greater numbers of individuals. It is also hoped that the findings will influence policies on self-sampling, both nationally and internationally. Lessons learnt from this study may be used to tailor and optimise self-testing delivery packages and models; drive demand-generation for diagnosis and self-testing in Georgia, the wider region and other countries around the world; and support the gaining of market approval for self-testing devices in jurisdictions where self-tests remain unregulated.

Operational lessons learnt from our COVID-19 self-testing pilot, however, must be considered in context for other diseases that are more stigmatised, such as HIV, where self-tests have the capacity to decrease the gap in testing [36], but, for example, reporting mechanisms for individuals to disclose their infection status might need to be adapted. Another mixed-methods study, also conducted in Georgia, to investigate self-testing for HCV among populations at increased risk of this infection, such as people who inject drugs, men who have sex with men, and transgender people, again found that people considered self-testing very convenient and easy to use [37].

Our study had some limitations. These include the potential bias towards acceptability of the implementation or increased satisfaction with self-tests, as study staff were also considered participants. However, there were only 65 study staff who were also participants, corresponding to just 3% of the total number of participants. Further limitations included the risk of memory bias, social desirability and observer bias in the interviews. Another potential limitation of our study was the presence of inconsistencies in the ID numbers within the dataset. While data manipulation procedures were instrumental in ensuring dataset integrity, it was not feasible to correct all ID numbers due to the number of initial inconsistencies. Despite this limitation, the remaining dataset contained all responses per timepoint and provided valuable insights for our analysis. Finally, there could have been some limitations due to the self-test specificities and sensitivities and potential false-negative results in asymptomatic individuals, or issues with individuals’ errors in the use of self-tests and the interpretation of results [38, 39]. However, RADTs have been shown to have high sensitivity and excellent specificity [8, 40]. Nevertheless, to assign a diagnosis of COVID-19, the interpretation of self-test results must be considered in combination with clinical information and according to updated national guidelines. Despite some potential limitations, RADTs for SARS-CoV-2are recommended by WHO to be offered as self-tests, due to the evidence in support of users being able to reliably and accurately self-test and because they reduce inequalities in access to testing [41]. Inherent to the study design, a limitation of the study was that we were not able to obtain socio-demographic information from those who did not answer the to compare their data with the ones who responded.

Strengths of our study include that the design was sufficiently flexible to be adapted to the study’s needs, the results were linked to the existing national COVID-19 surveillance database, trust was built among stakeholders, staff at sites and participants, and that a mobile application was launched nationwide to facilitate the reporting of self-test results.

While we understand that there is a gender skew among participants in this study, following global trends where women constitute 70% of the healthcare and education sectors, it is important to learn from this implementation and tailor approaches (trainings, workshops, sensitization materials) to reach other workplaces and population groups, including children and adolescents. The findings of our study have implications for the broader adoption of self-testing in diverse settings and beyond COVID-19 and can guide the operational aspects of introducing and scaling up self-testing for various diseases within the community. Future research should focus on evaluating the long-term sustainability and cost-effectiveness of self-testing programmes, while also exploring strategies to further enhance uptake of and adherence to self-testing initiatives for various diseases and to bridge the diagnostic gap.

## Conclusions

This study has produced valuable evidence regarding the feasibility and acceptability of self-testing in workplace settings and as part of a national testing programme for groups at high risk of infections, which subsequently informed the successful scale-up of COVID-19 self-testing in various healthcare centres across different regions of Georgia. Enrolment and participation rates in the COVID-19 self-testing pilot study were high, with consistent weekly reporting over a six-month period. This pilot study successfully detected more than 600 COVID-19 cases, half of which were identified among household members. Self-testing increased participants’ perception of safety. After implementation, there was a slight increase in individuals’ willingness to perform COVID-19 self-tests and report the results, and people’s knowledge of self-testing increased. Participants expressed a high degree of satisfaction with the use of self-testing, especially those residing in remote areas who no longer needed to travel long distances for diagnosis. Notably, self-testing greatly improved access to testing for teaching staff and their household members in rural villages.

In Georgia, this pilot study has improved pandemic preparedness and strengthened capabilities to incorporate self-testing for other diseases through the expansion of the national self-testing reporting system, the development of self-testing communications materials, changes in the national legal framework, the establishment of self-testing coordination mechanisms within sites and within NCDC, and by changing perceptions around self-testing and self-care, both among study participants and national stakeholders. This research contributes to the evidence on the use of self-testing strategies in workplaces for staff at high risk of exposure and in remote locations and highlights the importance of secondary distribution. Lessons learnt from this study have the potential to inform operational aspects of the introduction and scale-up of self-testing for other diseases during health emergencies or routine care, in other countries and settings, particularly resource-limited settings.

### Supplementary Information

**Additional file 1: Annex I.** Structured questionnaire administered at baseline, mid-point and end-point. **Annex II.** Knowledge and satisfaction survey. **Annex III.** COREQ (COnsolidated criteria for REporting Qualitative research) Checklist. **Annex IV.** Uptake of COVID-19 self-tests, reported to the national database, by type of user, gender and site. **Annex V.** Uptake of COVID-19 self-tests, reported to the national database, by type of user, self-test result, and site. **Annex VI.** Trends and potential associations among COVID-19 perceptions and willingness to self-test and report results, by time point and sites. **Annex VII.** Analysis of variance (ANOVA) among COVID-19 perceptions and willingness to self-test and report results, by time points and sites. **Annex VIII.** Socio-demographic characteristics of participants interviewed in the semi-structured interviews. **Annex IX.** Coding tree for the COVID-19 self-testing pilot in Georgia based on semi-structured interviews: main theme, sub-themes and codes.

## Data Availability

The datasets used and/or analysed during the current study are available from the corresponding author on reasonable request.
