# Supplementary material for: Implementing a pilot study of COVID-19 self-testing in high-risk populations and remote locations: results and lessons learnt
Source: BMC Public Health. 2024 Feb 17;24:511. doi: 10.1186/s12889-024-17930-2 (PMC10874527; doi:10.1186/s12889-024-17930-2)
Supplement: Supplementary file 1 — Additional file 1: Annex I. Structured questionnaire administered at baseline, mid-point and end-point. Annex II. Knowledge and satisfaction survey. Annex III. COREQ (COnsolidated criteria for REporting Qualitative research) Checklist. Annex IV. Uptake of COVID-19 self-tests, reported to the national database, by type of user, gender and site. Annex V. Uptake of COVID-19 self-tests, reported to the national database, by type of user, self-test result, and site. Annex VI. Trends and potential associations among COVID-19 perceptions and willingness to self-test and report results, by time point and sites. Annex VII. Analysis of variance (ANOVA) among COVID-19 perceptions and willingness to self-test and report results, by time points and sites. Annex VIII. Socio-demographic characteristics of participants interviewed in the semi-structured interviews. Annex IX. Coding tree for the COVID-19 self-testing pilot in Georgia based on semi-structured interviews: main theme, sub-themes and codes. [file 12889_2024_17930_MOESM1_ESM.docx]

**Supporting information**

**Annex I: Structured questionnaire administered at baseline, mid-point and end-point**

**Annex II: Knowledge and satisfaction survey**

The following questionnaire was administered at enrolment. The same survey was administered again at the mid- and end-points, but without the demographic and medical history questions.

**Online questionnaire:**

1. **Participant pilot ID** [ GCS01 - _ _ - F _ _ - _ _ ]

**Demographic data**

1. **What is your age?** [list of numbers from 14 to 95]
2. **What is your gender?**

- Male
- Female
- Prefer not to say

1. **What is your highest level of education obtained?**

- Elementary school
- Secondary school (9th grade)
- High school
- Professional technical education
- University (bachelor’s or master’s degree)
- University (PhD)

1. **What is your occupation? Please select one option.**

- Teacher
- Director
- Maintenance staff
- Cleaner
- Librarian
- Medical doctor
- Nurse
- Sanitarian
- Laboratory personnel
- Administration
- Cook
- Driver
- Other _______

1. **How many people live in your household, not including yourself?** [list of numbers from 0 to 15]
2. [If Q6 is different to “0”], **How many people in your household were employed/paid for work in the past 3 months,** **not including yourself?** [list of numbers from 0 to 15]
3. **How many children (aged 12 years or older) are there in your household?** [list of numbers from 0 to 15]
4. **Do you own a smartphone?** Single choice [Yes, No]

**Medical history (potential COVID-19 risks)**

1. **Are you vaccinated against COVID-19? Please select one option.**
   - Yes, I received one dose
   - Yes, I received two doses
   - Yes, I received three or more doses
   - No, I did not receive any doses
2. **Do you have any of these conditions? Please select all that apply.**
   - Chronic conditions (cancer; heart condition, stroke, or cerebrovascular disease; immunocompromised condition; cystic fibrosis; chronic lung, kidney, or liver disease; transplant etc.)
   - Infections (HIV, tuberculosis, others)
   - Diabetes, overweight, physical inactivity
   - Mental health/neurological conditions (schizophrenia spectrum disorder, depression, dementia, others)
   - Disabilities
   - Pregnancy
   - Smoking (current or former)
   - Substance use disorder
   - None
3. **Have you been diagnosed with COVID-19 before?**
   - Yes
   - No
   - Don’t know
   - Don’t remember
4. **If so, how severe was your experience of COVID-19?**
   - Asymptomatic
   - Flu-like symptoms
   - Mild to moderate symptoms
   - Severe symptoms (respiratory distress, needed hospital admission)

**Perceptions and satisfaction with regards to COVID-19 self-testing**

1. **How much do you agree with the following sentence “Currently, I am worried about the COVID-19 situation”?** [Strongly agree, agree, neutral, disagree, strongly disagree]
2. **How much do you agree with the following sentence: “I think I will perform COVID-19 self-tests weekly for the next few months, as part of the pilot study”?** [Strongly agree, agree, neutral, disagree, strongly disagree]
3. **How much do you agree with the following sentence: “I think I will report COVID-19 self-test results weekly for the next few months, as part of the pilot study”?** [Strongly agree, agree, neutral, disagree, strongly disagree]
4. **How much do you agree with the following sentence: “I understand the benefits related to my workplace of self-testing for COVID-19”?** [Strongly agree, agree, neutral, disagree, strongly disagree]
5. **What benefits do you see in this self-testing model? Please select all that apply.**
   - Self-test at home for weekly mandatory testing
   - Self-test at home if I am symptomatic/had a close contact
   - Provide a self-test for my household members
   - I don’t see any benefits compared with other COVID-19 diagnostics
   - Other (please specify): ____________________
6. **Which of these options do you prefer for weekly COVID-19 testing?**
   - Being tested by a professional at my workplace (professional antigen test)
   - Being tested by a professional at a healthcare facility (professional antigen test)
   - Being tested by myself at home (self-test used, as in the pilot study)
   - Being tested by myself at my workplace (self-test used, as in the pilot study)
   - I don’t know
   - Other (please specify): ___________________

**Knowledge**

1. **Where should you take the sample for COVID-19 self-testing?**
   - In one nostril, no more than 2 cm deep
   - In two nostrils, no more than 2 cm deep
   - In the nose, more than 2 cm deep
   - In the nose, it does not matter how deep, but in circles
   - In the mouth and nose
2. **What does a positive COVID-19 self-test result mean?**
   - I am not infected with COVID-19
   - I had COVID-19 in the past
   - I have a high likelihood of developing severe symptoms
   - I am likely to be infected with COVID-19
   - I don’t know
3. **What do you need to do if you have a positive COVID-19 self-test result? Please select all that apply.**
   - Check the latest national guidelines
   - Self-isolate as much as possible
   - Report the results immediately
   - Call my close contacts
   - Go to work wearing a mask and maintain hygiene measures
4. **What does a faint line in the COVID-19 self-test mean?**

- I might have COVID-19 disease and can infect others
- I need to repeat the COVID-19 self-test
- It is a false-positive result
- I don’t have COVID-19
- I have COVID-19 but cannot infect others

1. **If you are negative for COVID-19 but have symptoms, what do you need to do? Please select all that apply.**

- Repeat the self-test immediately
- Repeat self-tests for a maximum of five consecutive days
- See if your symptoms get worse, and if they do consult a healthcare centre
- Go to work and wear a mask, you don’t need to test again
- I don’t know

**Annex II: Semi-structured interview guide**

| GCS01 - Site ID: _ _ GCS01- _ _ - F _ _ - _ _ Facilitator ID: F _ _ |
| --- |
| Opening statement:  Thank you for agreeing to take part in this interview today. We will be discussing your experiences with COVID-19 self-testing and your views on how self-testing should be provided.  Topics to be explored:  The topics that we will be discussing today include your perceptions and experiences of regularly using self-tests, potential barriers to self-testing, factors that could facilitate/motivate you to perform self-testing, and behaviours after receiving a positive result. |
| Perceptions and experiences of COVID-19 self-testing   - How would you describe your experience during the pilot study? Did you experience any difficulties while performing self-testing? How did you overcome these difficulties? - How do you report your results? - Are you self-testing regularly? Every week? Do you think your colleagues at work are testing regularly? Why do you think they are/are not doing so? - Do you think that your satisfaction with self-testing has changed from the beginning of the pilot study until now? In what sense? (e.g., your confidence in performing the test, recommending it to others, etc.) - What do you think about allowing people to self-test for COVID-19? What do you think are the main advantages? What are the main disadvantages? - Do you think other people would be interested in self-testing? Why or why not? - What do you think about self-testing? Did you do it before participating in the pilot study? Was it easy/difficult? - Did you perform your COVID-19 self-test every week? Why? How did you feel? - Did you have a positive result? What did you do? Did you know what you had to do? Did your household members use a self-test? How did you feel about the diagnosis? - Did you have any invalid results? What did you do? How did it make you feel? - What is the thing you like the most about this testing system? What do you like the least? - How do you feel about going to work now, compared with a few months ago (e.g., safer, stressed, overwhelmed, calm…)? How does your family at home feel now (e.g., safer, stressed, overwhelmed, calm...)? - If you could share some tips with a person who is not very confident about self-testing, what would you tell them? - Would you use a COVID-19 self-test again in the future (not for the workplace)? Why or why not? - What do you think are the main reasons why people choose to use COVID-19 self-testing? Which groups of people? Or people in which circumstances? |
| Other questions: Do you have any questions/concerns that we have not addressed, that you would like to share? Do you have anything else to add? |
| Thank you for your time and participation. We have learned a lot from our discussion here today, and we hope this time has also been useful for you. |

**Annex III: COREQ (COnsolidated criteria for REporting Qualitative research) Checklist**

A checklist of items that should be included in reports of qualitative research. You must report the page number in your manuscript where you consider each of the items listed in this checklist. If you have not included this information, either revise your manuscript accordingly before submitting or note N/A.

| **Topic** | **Item No.** | **Guide Questions/Description** | **Reported on Page No.** |
| --- | --- | --- | --- |
| **Domain 1: Research team and reﬂexivity** | | | |
| *Personal characteristics* | | | |
| Interviewer/facilitator | 1 | Which author/s conducted the interview or focus group? | 15 |
| Credentials | 2 | What were the researcher’s credentials? E.g. PhD, MD | 15 |
| Occupation | 3 | What was their occupation at the time of the study? | 15 |
| Gender | 4 | Was the researcher male or female? | 15 |
| Experience and training | 5 | What experience or training did the researcher have? | 15 |
| *Relationship with participants* | | | |
| Relationship established | 6 | Was a relationship established prior to study commencement? | 15 |
| Participant knowledge of  the interviewer | 7 | What did the participants know about the researcher? e.g. personal  goals, reasons for doing the research | 15 |
| Interviewer characteristics | 8 | What characteristics were reported about the inter viewer/facilitator?  e.g. Bias, assumptions, reasons and interests in the research topic | 15 |
| **Domain 2: Study design** | | | |
| *Theoretical framework* | | | |
| Methodological orientation and Theory | 9 | What methodological orientation was stated to underpin the study? e.g. grounded theory, discourse analysis, ethnography, phenomenology,  content analysis | 16 |
| *Participant selection* | | | |
| Sampling | 10 | How were participants selected? e.g. purposive, convenience,  consecutive, snowball | 10 |
| Method of approach | 11 | How were participants approached? e.g. face-to-face, telephone, mail,  email | 10 |
| Sample size | 12 | How many participants were in the study? | 26 |
| Non-participation | 13 | How many people refused to participate or dropped out? Reasons? | NA |
| *Setting* | | | |
| Setting of data collection | 14 | Where was the data collected? e.g. home, clinic, workplace | 14 |
| Presence of non-  participants | 15 | Was anyone else present besides the participants and researchers? | 15 |
| Description of sample | 16 | What are the important characteristics of the sample? e.g. demographic data, date | 26 |
| *Data collection* | | | |
| Interview guide | 17 | Were questions, prompts, guides provided by the authors? Was it pilot  tested? | 15 |
| Repeat interviews | 18 | Were repeat inter views carried out? If yes, how many? | 15 |
| Audio/visual recording | 19 | Did the research use audio or visual recording to collect the data? | 16 |
| Field notes | 20 | Were field notes made during and/or after the inter view or focus group? | 15 |
| Duration | 21 | What was the duration of the inter views or focus group? | 15 |
| Data saturation | 22 | Was data saturation discussed? | 14 |
| Transcripts returned | 23 | Were transcripts returned to participants for comment and/or | NA |
| **Domain 3: analysis and ﬁndings** | | | |
| *Data analysis* | | | |
| Number of data coders | 24 | How many data coders coded the data? | NA |
| Description of the coding tree | 25 | Did authors provide a description of the coding tree? | 27 |
| Derivation of themes | 26 | Were themes identified in advance or derived from the data? | 27 |
| Software | 27 | What software, if applicable, was used to manage the data? | NA |
| Participant checking | 28 | Did participants provide feedback on the findings? | NA |
| *Reporting* | | | |
| Quotations presented | 29 | Were participant quotations presented to illustrate the themes/findings? Was each quotation identified? e.g. participant number | 27-29 |
| Data and findings consistent | 30 | Was there consistency between the data presented and the findings? | 28-29 |
| Clarity of major themes | 31 | Were major themes clearly presented in the findings? | 27 |
| Clarity of minor themes | 32 | Is there a description of diverse cases or discussion of minor themes? | 27-29 |

Developed from: Tong A, Sainsbury P, Craig J. Consolidated criteria for reporting qualitative research (COREQ): a 32-item checklist for interviews and focus groups. *International Journal for Quality in Health Care*. 2007. Volume 19, Number 6: pp. 349 – 357

**Annex IV: Uptake of COVID-19 self-tests, reported to the national database, by type of user, gender and site**

| Type of user | Gender | Healthcare centre | | Nursing home | | School | | Test |
| --- | --- | --- | --- | --- | --- | --- | --- | --- |
| Participants | Sub-total | 35253 | | 3120 | | 13527 | | χ2=95.164* |
|  | Female | 27533 | 78.1% | 2666 | 85.4% | 10730 | 79.3% |  |
|  | Male | 7720 | 21.9% | 454 | 14.6% | 2797 | 20.7% |  |
| Household members | Sub-total | 1023 | | 14 | | 37 | | χ2 =0.767 |
|  | Female | 484 | 47.3% | 5 | 35.7% | 17 | 45.9% |  |
|  | Male | 539 | 52.7% | 9 | 64.3% | 20 | 54.1% |  |

* Significant result

**Annex V: Uptake of COVID-19 self-tests, reported to the national database, by type of user, self-test result, and site**

| Type of user | Self-test result | Healthcare centre | | Nursing home | | School | | Test |
| --- | --- | --- | --- | --- | --- | --- | --- | --- |
| Participants | Sub-totals | 35253 | | 3120 | | 13527 | | χ2 =71.379*** |
|  | **Invalid** | 26 | 0.1% | 10 | 0.3% | 18 | 0.1% |  |
|  | **Negative** | 34975 | 99.2% | 3100 | 99.4% | 13484 | 99.7% |  |
|  | **Positive** | 252 | 0.7% | 10 | 0.3% | 25 | 0.2% |  |
| Household members | Sub-totals | 1023 | | 14 | | 37 | | χ2 =15.77* |
|  | **Invalid** | 6 | 0.6% | 0 | 0% | 0 | 0% |  |
|  | **Negative** | 716 | 70% | 5 | 35.7% | 18 | 48.6% |  |
|  | **Positive** | 301 | 29.4% | 9 | 64.3% | 19 | 51.4% |  |

* Significant result

**Annex VI: Trends and potential associations among COVID-19 perceptions and willingness to self-test and report results, by time point and sites**
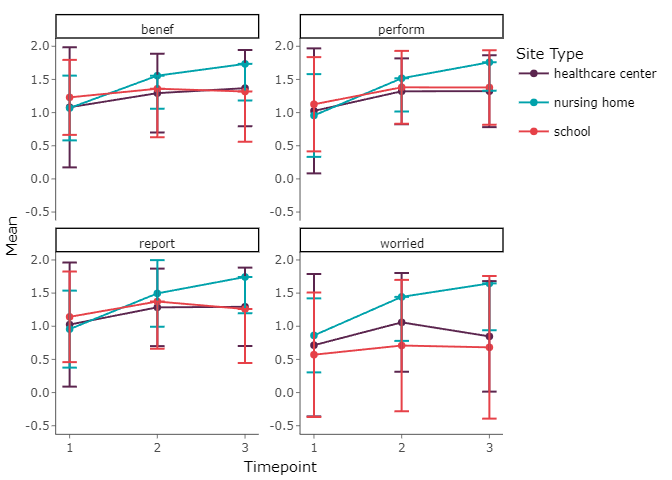


**Annex VII: Analysis of variance (ANOVA) among COVID-19 perceptions and willingness to self-test and report results, by time points and sites**

|  | Question | Factors | Df | Sum Sq | Mean Sq | F value | Pr(>F) | P.ADJUST |
| --- | --- | --- | --- | --- | --- | --- | --- | --- |
| 1 | **Worry** | **Site Type** | **2** | **119.8644** | **59.93218** | **73.67428** | **3.48E-32** | **3.48E-31** |
| 2 | **Worry** | **Time** | **1** | **19.57748** | **19.57748** | **24.06648** | **9.65E-07** | **2.14E-06** |
| 3 | **Worry** | **Gender** | **2** | **2.555859** | **1.277929** | **1.570951** | **0.207967** | **0.207967** |
| 4 | **Worry** | **Age** | **1** | **41.71007** | **41.71007** | **51.27395** | **9.41E-13** | **3.76E-12** |
| 5 | **Worry** | **Interaction: Site Type x Time** | **2** | **22.42124** | **11.21062** | **13.78115** | **1.08E-06** | **2.16E-06** |
| 6 | **Worry** | **Residuals** | **4282** | **3483.3** | **0.813475** |  |  |  |
| 7 | **Perform** | **Site Type** | **2** | **10.44478** | **5.222389** | **12.40589** | **4.25E-06** | **7.08E-06** |
| 8 | **Perform** | **Time** | **1** | **71.26962** | **71.26962** | **169.3024** | **5.58E-38** | **1.12E-36** |
| 9 | **Perform** | **Gender** | **2** | **4.135867** | **2.067934** | **4.912417** | **0.007397** | **0.009246** |
| 10 | **Perform** | **Age** | **1** | **3.073171** | **3.073171** | **7.300376** | **0.006922** | **0.009229** |
| 11 | **Perform** | **Interaction: Site Type x Time** | **2** | **14.96587** | **7.482933** | **17.77585** | **2.05E-08** | **5.86E-08** |
| 12 | **Perform** | **Residuals** | **4230** | **1780.663** | **0.420961** |  |  |  |
| 13 | **Benefits** | **Site Type** | **2** | **11.90473** | **5.952365** | **12.82886** | **2.79E-06** | **5.07E-06** |
| 14 | **Benefits** | **Time** | **1** | **47.9148** | **47.9148** | **103.2686** | **5.47E-24** | **3.64E-23** |
| 15 | **Benefits** | **Gender** | **2** | **2.22094** | **1.11047** | **2.393345** | **0.091446** | **0.107583** |
| 16 | **Benefits** | **Age** | **1** | **4.42622** | **4.42622** | **9.539631** | **0.002024** | **0.002891** |
| 17 | **Benefits** | **Interaction: Site Type x Time** | **2** | **15.61719** | **7.808597** | **16.82951** | **5.24E-08** | **1.31E-07** |
| 18 | **Benefits** | **Residuals** | **4282** | **1986.772** | **0.463982** |  |  |  |
| 19 | **Report** | **Site Type** | **2** | **10.89006** | **5.44503** | **10.8721** | **1.95E-05** | **3E-05** |
| 20 | **Report** | **Time** | **1** | **48.47219** | **48.47219** | **96.78449** | **1.34E-22** | **6.70E-22** |
| 21 | **Report** | **Gender** | **2** | **1.749062** | **0.874531** | **1.746178** | **0.174564** | **0.183751** |
| 22 | **Report** | **Age** | **1** | **1.381297** | **1.381297** | **2.758039** | **0.09684** | **0.1076** |
| 23 | **Report** | **Interaction: Site Type x Time** | **2** | **20.17018** | **10.08509** | **20.13692** | **1.97E-09** | **6.58E-09** |
| 24 | **Report** | **Residuals** | **4282** | **2144.537** | **0.500826** |  |  |  |

**Annex VIII: Socio-demographic characteristics of participants interviewed in the semi-structured interviews**

| Variable | Category | Mid-point  N=32 | End-point  N=22 |
| --- | --- | --- | --- |
|  | | **n (%)** | **n (%)** |
| Age | ≤ 30 | 6 (19%) | 2 (9%) |
|  | 31–40 | 5 (16%) | 6 (27%) |
|  | 41–50 | 12 (38%) | 7 (32%) |
|  | ≥51 | 9 (28%) | 7 (32%) |
| Gender | Female | 29 (91%) | 18 (82%) |
|  | Male | 3 (9%) | 4 (18%) |
| Site | Hospital or clinic | 19 (59%) | 16 (73%) |
|  | Nursing home | 5 (16%) | 5 (23%) |
|  | School | 8 (25%) | 1 (5%) |
| Occupation | Medical doctor | 8 (25%) | 3 (14%) |
|  | Nurse | 3 (9%) | 5 (23%) |
|  | Caregiver | 3 (9%) | 0 (0%) |
|  | Administration, management or quality service | 6 (19%) | 6 (27%) |
|  | Director | 2 (6%) | 0 (0%) |
|  | Teacher | 4 (13%) | 1 (5%) |
|  | Other (cleaner, kitchen staff, security, lawyer, economist, logistics department…) | 6 (19%) | 7 (32%) |
| Study staff | Yes | 0 | 6 (27%) |

**Annex IV: Coding tree for the COVID-19 self-testing pilot in Georgia based on semi-structured interviews: main theme, sub-themes and codes**

| **COVID-19 self-testing experiences** |
| --- |
|  |
| **1: Previous COVID-19 experiences** |
| 1: Personal |
| 2: Household members |
| 3: Positive |
| 4: Self-testing experiences |
| **2: Pilot COVID-19 self-testing experiences** |
| 5: Use |
| 6: Positive |
| 7: Means of reporting |
| 8: Household members' experiences |
| 9: Colleagues' experiences |
| 10: Changing experiences after use |
| **3: Advantages** |
| 11: Convenience |
| 12: Easy |
| 13: Time saver |
| **4: Disadvantages** |
| 14: None |
| 15: Late |
| 16: Long instructions |
| **5: Feelings** |
| 17: Positive feelings (happy) |
| 18: Safe |
| 19: Confident |
| **6: Willingness to pay** |
| 20: Cheap |
| 21: Vulnerable populations |
| 22: General population |
